# Overweight across the life course and adipokines, inflammatory and endothelial markers at age 60–64 years: evidence from the 1946 birth cohort

**DOI:** 10.1038/ijo.2015.19

**Published:** 2015-06-09

**Authors:** E T Murray, R Hardy, A Hughes, A Wills, N Sattar, J Deanfield, D Kuh, P Whincup, and the NSHD Scientific and Data Collection team

**Affiliations:** 1 Population Health Research Institute, St George’s University of London, London, UK; 2 MRC Unit for Lifelong Health and Ageing at University College London, London, UK; 3 Institute of Cardiovascular Science, University College London, London, UK; 4 School of Clinical Sciences, University of Bristol, Bristol, UK; 5 British Heart Foundation Glasgow Cardiovascular Research Centre, University of Glasgow, Glasgow, UK; 6 National Centre for Cardiovascular Prevention and Outcomes, University College London, London, UK

## Abstract

**Background/Objectives:**

There is growing evidence that early development of obesity increases cardiovascular risk later in life, but less is known about whether there are effects of long-term excess body weight on the biological drivers associated with the atherosclerotic pathway, particularly adipokines, inflammatory and endothelial markers. This paper therefore investigates the influence of overweight across the life course on levels of these markers at retirement age.

**Subjects/Methods:**

Data from the Medical Research Council National Survey of Health and Development (*n*=1784) were used to examine the associations between overweight status at 2, 4, 6, 7, 11, 15, 20, 26, 36, 43, 53 and 60–64 years (body mass index (BMI)⩾25 kg m^−2^ for adult ages and gender-specific cut-points for childhood ages equivalent to BMI⩾25 kg m^−2^) and measurements of adipokines (leptin and adiponectin), inflammatory markers (C-reactive protein (CRP), interleukin-6 (IL-6)) and endothelial markers (E-selectin, tissue plasminogen activator (t-PA) and von Willebrand factor) at 60–64 years. In addition, the fit of different life course models (sensitive periods/accumulation) were compared using partial F-tests.

**Results:**

In age- and sex-adjusted models, overweight at 11 years and onwards was associated with higher leptin, CRP and IL-6 and lower adiponectin; overweight at 15 years and onwards was associated with higher E-selectin and t-PA. Associations between overweight at all ages earlier than 60–64 with leptin, adiponectin, CRP and IL-6 were reduced but remained apparent after adjustment for overweight at 60–64 years; whereas those with E-selectin and t-PA were entirely explained. An accumulation model best described the associations between overweight across the life course with adipokines and inflammatory markers, whereas for the endothelial markers, the sensitive period model for 60–64 years provided a slightly better fit than the accumulation model.

**Conclusions:**

Overweight across the life course has a cumulative influence on adipokines, inflammatory and possibly endothelial markers. Avoidance of overweight from adolescence onwards is likely important for cardiovascular disease prevention.

## Introduction

Overweight and obesity represent a major global health problem; the World Health Organization estimated that in 2008, more than 1.4 billion adults were overweight (body mass index (BMI) 25–29 kg m^−2^) and 500 million obese (BMI>30 kg m^−2^) globally.^1^ It has been calculated that if all persons were not overweight or obese, ~3.4 million (6.4%) of all deaths annually,^2^ and approximately a quarter of all deaths due to ischaemic heart disease could be prevented;^1^ the largest single cause of death.^3^

In recent years, the mechanism(s) by which excess body weight produces adverse cardiovascular outcomes has attracted interest, with one potential pathway being the dysfunction of adipose tissue.^4, 5^ When energy balance is positive, more calories are consumed than expended, adipocytes fill up with fatty acids, and patterns of adipokine secretion are altered; leptin secretion rises and adiponectin declines. The rising concentration of leptin is associated with higher levels of inflammatory cytokines, which have been implicated in localised inflammation and facilitate cholesterol accumulation (hallmarks of the atherosclerotic process). Lower adiponectin levels may lead to an impaired ability to repair endothelial damage and inhibit the endothelial inflammatory response, but this is an area of ongoing research.^4, 5, 6, 7^

A notable feature of the global obesity epidemic is the appearance of overweight and obesity in childhood and early adulthood.^8^ There is growing evidence that the early development of obesity increases cardiovascular risk later in life,^9, 10, 11^ but less is known about whether there are persistent effects of long-term excess body weight on the biological drivers associated with the atherosclerotic pathway, particularly adipokines, inflammatory and endothelial markers, which have collectively been referred to as ‘adiposopathic markers’^7^ because their secretion is associated with adipose tissue hypertrophy. Numerous studies have documented cross-sectional associations between higher levels of adiposity and adverse levels of these ‘adiposopathic’ markers, in mid to late adulthood,^12, 13^ with relatively short-term changes in weight altering the levels of these factors.^14, 15, 16, 17, 18^ A few studies have shown that weight gain from adolescence to young adulthood, and from early to mid-adulthood, are associated with higher levels of inflammatory marker C-reactive protein (CRP) at ages 18, (ref.^19^) 22 (ref.^20^) and 31 years.^21^ However, it is not clear how much adiposity levels before middle age affect levels of adiposopathic markers in later life, and whether any influence reflects a cumulative process or the influence of adiposity at specific sensitive periods. If it can also be shown that levels of other inflammatory markers, plus adipokines and endothelial markers, are associated with excess body weight at similar ages in the life course, this would give additional support to the hypothesis that adipose tissue dysfunction is one of the pathways by which overweight and obesity lead to cardiovascular disease (CVD).

We have therefore used data from the Medical Research Council National Survey of Health and Development (NSHD) to explore at what stage in the life course overweight is associated with adiposopathic markers of adipokines (leptin and adiponectin), inflammatory (CRP, interleukin-6 (IL-6) and endothelial markers (E-selectin, tissue plasminogen activator (t-PA) and von Willebrand factor (vWF)) measured in early old age (60–64 years). Our aims were to: (1) determine how early in the life course being overweight is associated with each adiposopathic marker; (2) identify whether being overweight at a particular age, or at multiple ages (particularly including adolescence, early adult and later adult), best describes the relationship between overweight across life and each adiposopathic marker; and (3) given the findings of aim 2, whether associations of the chosen model are independent of potential confounders of smoking, childhood social class and adult social class. We have treated overweight as a dichotomous variable, to facilitate the life course analytic approach used in this paper, and to define the specific impact of overweight as commonly defined (BMI⩾25 kg m^−2^).

## Materials and methods

### Study design

The Medical Research Council NSHD is a socially stratified British cohort of individuals who have been followed up 23 times between birth and late middle age.^22^ The original cohort comprised 5362 singleton children born during 1 week in March 1946. At the most recent clinic assessment at 60–64 years (2006–2010), eligible cohort members (those known to be alive and with a known address in England, Scotland or Wales) were invited for an assessment at one of the six clinical research facilities (CRFs) or to be visited by a research nurse at home. Invitations were not sent to those who had died (*n*=778, 14.5% of the original cohort), who had emigrated (570, 10.6%), who were lost to follow-up (594, 11.1%) and who had previously withdrawn from the study (564, 10.5%). Of the 2856 cohort members invited, 1690 (59.2%) attended a CRF for full examination and 539 (18.9%) were examined at home by a trained nurse.^23^ Ethical approval was given by the Central Manchester Research Ethics Committee for genetic studies of lifelong health, disease and ageing (07/H1008/168) and the Scottish A Research Ethics Committee.

### Life course BMI

Heights (m) and weights (kg) were measured by trained observers in a standardised manner at 2, 4, 6, 7, 11, 15, 36, 43, 53 and 60–64 years, and were self-reported by cohort members at 20 and 26 years. BMI was calculated (kg m^−2^) at each age, with ‘overweight’ defined according to the World Health Organization guidelines as a BMI⩾25 kg m^−2^ for adult ages and gender-specific cut-points for childhood ages (2–15 years) equivalent to a BMI⩾25 kg m^−2^ at age 18 years.^24^

### Measurement of adiposopathic markers at 60–64 years

Overnight fasting blood samples were taken during the clinic or home visit and initially processed at the CRF laboratories. Aliquots were frozen and stored before being transferred to the Medical Research Council Human Nutrition Research Laboratory in Cambridge where analyses of inflammatory marker CRP was processed according to standardised protocols. Analyses of adipokines (leptin and adiponectin), endothelial markers (E-selectin and t-PA) and inflammatory marker IL-6 were undertaken by British Heart Foundation Research Centre in Glasgow. The method and commercial assay, plus interassay coefficients of variations are given in Supplementary Table S1. Age at measurement in months was recorded.

### Covariates

Information on smoking status, occupational social class and disease conditions were obtained by questionnaire at 60–64 years; cigarette smoking was classified into three categories: never, former and current. Father’s occupation when the cohort member was age 4 years and own occupational class at age 53 years were chosen to represent childhood and adult socioeconomic positions, respectively. The Registrar General’s six-group classification^25^ was collapsed into four groups: professional and intermediate (I and II); skilled non-manual (IIInm); skilled manual (IIIm); and semiskilled and unskilled manual (IV and V). Wherever possible, missing values were imputed from adjacent ages (33 values from age 11 and 14 values from age 15 for childhood social class; 107 values from age 36 for early adulthood; 107 values from age 43 for late adulthood). Stroke and CVD diagnoses (angina or myocardial infarction) were based on participant responses indicating at age 53 or 60–64 years whether a doctor had ever diagnosed them with the condition.^26^

### Statistical analysis

Analyses were undertaken using STATA version 12 (STATA corp LP, TX, USA). All adipokines, endothelial and inflammatory markers were positively skewed and therefore transformed using the natural logarithm to achieve normality of their distributions. This analysis comprises three parts aimed at investigating when in the life course excessive body weight may explain differences in the adiposipathic markers examined at the age of 60–64 years. First, age-adjusted models were fitted for each novel risk factor linearly regressed on BMI at each age (2, 4, 6, 7, 11, 15, 20, 26, 36, 43, 53 and 60–64 years) separately. Linearity of associations was assessed using scatterplots and Lowess smoothed curves. The same models were then fitted using dichotomised ‘overweight’ and ‘normal weight’ categories using established, worldwide definitions at each age. This dichotomous approach also facilitated the fitting of life course models, as described below. Each natural logarithm-transformed coefficient was multiplied by 100 to express all regression coefficients as mean percentage differences^27^ in each adiposopathic marker for overweight compared with non-overweight cohort members. Gender differences in the relationships between overweight at each age and each adiposipathic marker were investigated by testing interactions between sex and overweight and assessing associations separately for men and women. Each model was then adjusted for overweight status at 60–64 years in order to test the extent of mediation of earlier life overweight by later overweight. As few participants were classified as obese (>30 BMI) before age 43, sensitivity analyses were conducted by replacing the ‘overweight’ BMI cut-off value with the alternative level of 27.5 kg m^−2^ (and equivalent levels for childhood ages^24^). In addition, models were refitted as categorical variables comprising normal weight (<25 kg m^−2^), overweight (25–29.9) and obese (⩾30), at ages 43, 53 and 60–64.

Second, a structured modelling approach^28^ was used to examine different hypothesised life course overweight models in relation to each adiposopathic marker. The basic premise of the approach is to compare the model fit of a set of nested life course models with a saturated model containing all possible main effects and interactions. The original paper proposed the use of the F-test to compare the fit of each reduced model with the saturated model. A *P*-value that was not statistically significant (*P*>0.05) indicates that there is no evidence that the more complex model explained the data better than the simpler life course model. We also assessed model fit using Akaike Information Criteria (AIC), where a smaller AIC indicated a better model fit. To avoid zero cell counts in trajectory groups, and to reduce potential multi-colinearity of repeated overweight measurements (Supplementary Table S2), overweight categories at three ages were chosen to represent adolescence (15 years), mid-adulthood (36 years) and early old age (60–64 years) for this section of the analyses; resulting in eight possible trajectories. We did not include overweight status for childhood ages in life course models, as the prevalence of overweight before puberty was low in this cohort. In the main analysis, the life course models analysed were as follows: (1) sensitive period models for adolescence, mid-adulthood or early old age; and (2) accumulation of risk models of overweight in adolescence and mid-adulthood only, mid-adulthood and early old age only, and across all the three time periods. The three age accumulation models were then split further into a ‘relaxed’ accumulation model where associations of overweight at each age can have different strengths, whereas a ‘strict’ accumulation model indicated strengths of association were equal for all three ages. When there was evidence that two or more life course models for a given outcome provided as good a fit as the saturated model, we selected the model with the lowest AIC. When the null model fitted the data as well as the saturated model, the null model was selected. Sensitivity analysis was conducted separately replacing overweight at 15 with 20 years, age 36 with 26 or 43 years and age 60–64 with 53 years.

For the third part of the analyses, the best fitting life course model with adjustment for sex and age (in months) were then subsequently adjusted for smoking, and childhood and adulthood socioeconomic positions. To assess whether associations could be explained by disease status, all models were re-run after separate exclusion of prevalent CVD cases (*n*=151) and CRP levels indicative of acute infection (>10 mg l^−1^; *n*=107) at age 60–64 years. To investigate bias due to missing data, the main analysis was repeated with added imputed values from 50 imputed datasets obtained via chained equations, using 10 cycles per data set.^29^ The imputation model included all variables used in models (BMI at all ages, adiponectin, leptin, t-PA, E-selectin, vWF, CRP, IL-6, age, sex, CVD or stroke at age 60–64 years, smoking status 60–64 years, childhood socioeconomic position and adult socioeconomic position). Cohort members who had died before or during the most recent data collection (at age 60–64 years), or who were missing any adiposopathic markers, were excluded from the multiple imputation analysis.

## Results

Of the 2229 participants with a clinic or home visit at 60–64 years, 1784 (80.0%) had complete measurements for all seven adiposopathic markers. Those included were slightly younger in age at the time of assessment (63.2 vs 64.0 years), more likely to have attended the clinic than had a home visit (87.3% vs 29.7%), more likely to be in a non-manual childhood socioeconomic position (49.0% vs 43.6%) and adult socioeconomic position (72.6% vs 61.0%), and less likely to be overweight (⩾25 kg m^−2^) at 36 years (31.2% vs 36.6%), 53 years (69.5% vs 76.2%) and 63 years (69.5% vs 76.2% data not shown). Distribution of the adiposopathic markers and covariates at age 60–64 years, and the proportion of study members who were overweight at each age, are shown in Table 1, total and by gender.

**Figure 1:**
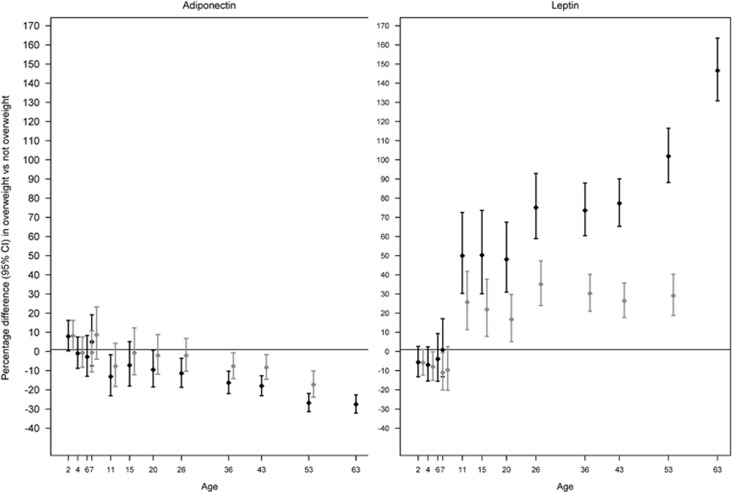
Percentage difference (95% CI) in adipokines at age 60–64 years for overweight (BMI>25 kg m^−2^) vs normal weight, fitted separately for each age. Black lines indicate adjustment for age and sex only. Grey lines indicate further adjustment for overweight status at age 60–64 years. CI, confidence interval.

**Figure 2:**
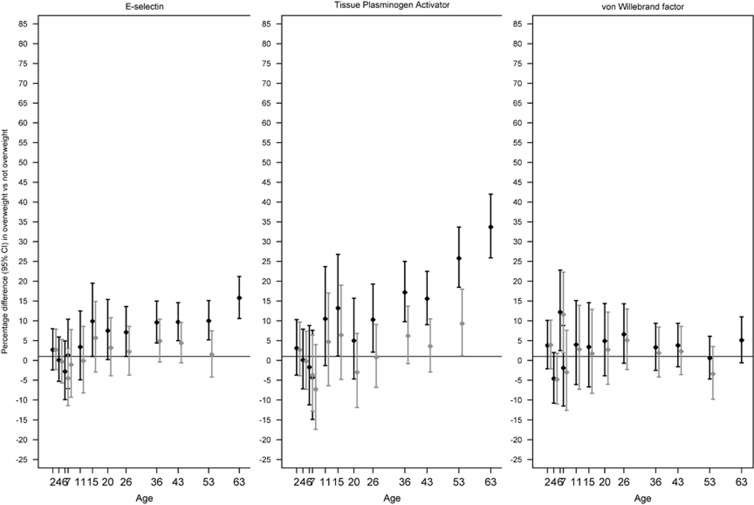
Percentage difference (95% CI) in endothelial makers at age 60–64 years for overweight (BMI>25 kg m^−2^) vs normal weight, fitted separately for each age. Black lines indicate adjustment for age and sex only. Grey lines indicate further adjustment for overweight status at age 60–64 years. CI, confidence interval.

**Figure 3:**
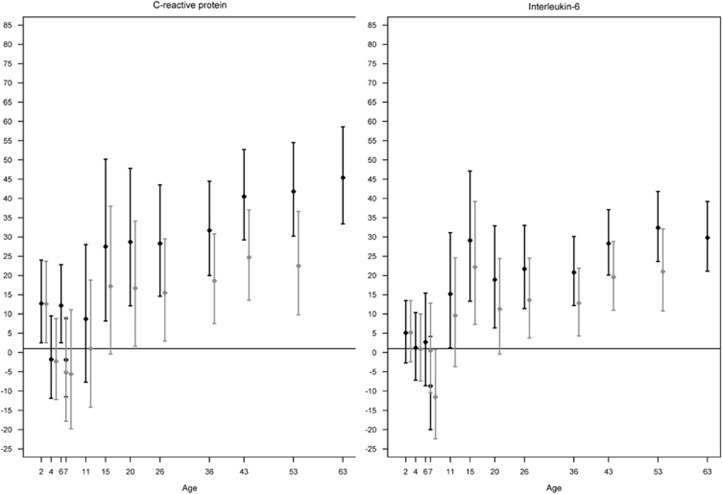
Percentage difference (95% CI) in inflammatory makers at age 60–64 years for overweight (BMI>25 kg m^−2^) vs normal weight, fitted separately for each age. Black lines indicate adjustment for age and sex only. Grey lines indicate further adjustment for overweight status at age 60–64 years. CI, confidence interval.

Figures 1, 2, 3 show age- and sex-adjusted models for overweight at each age, fitted separately, and each adiposopathic marker: the adipokines adiponectin and leptin in Figure 1, the endothelial markers E-selectin and t-PA in Figure 2 and the inflammatory markers CRP and IL-6 in Figure 3. Estimates and *P*-values are displayed separately in Supplementary Table S4. Greater BMI and overweight at 11 years and onwards tended to be associated with lower adiponectin and higher leptin, CRP and IL-6; higher BMI and overweight at 15 years and onwards tended to be associated with higher E-selectin and t-PA. Strengths of associations also tended to increase the older the age at which overweight was measured. For example, participants overweight at age 11 had mean leptin levels 50.0% (30.3–72.6%) higher than participants who were not overweight at age 11, whereas percentage differences had increased to 75.1% (58.9–92.9%) for overweight at age 26 years and to 146.6% (130.8–163.5%) at 60–64 years. Before the age of 11, the only association consistent with those at later ages was that between overweight at age 2 and higher CRP (12.7% (2.5–24.0%)). vWF was only associated with overweight at the age of 6 years, but not at later ages. The associations between overweight earlier than 60–64 years with leptin, adiponectin, CRP and IL-6 were reduced but remained apparent after adjustment for overweight at age 60–64 years; whereas E-selectin and t-PA were entirely explained. Results using BMI modelled as a continuous measure and those using the cut-off for overweight found similar associations, with the exception that associations of BMI and vWF were apparent at some adult ages when BMI was modelled as a continuous variable but not as a dichotomised variable (Supplementary Table S3).

Estimates using the alternative BMI cut-off of 27.5 kg m^−2^ were higher, but confidence intervals were wider (Supplementary Table S5). With the exception of vWF, models for ages 43, 53 and 60–64 years refitted as three categories of BMI, showed that relationships were dose related with both overweight and obese groups having mean adiponectin, leptin, CRP, IL-6, E-selectin and t-PA values worse than normal weight participants, but with effects stronger for obese participants (Supplementary Table S6).

Interaction tests were also carried out to examine whether the associations between being overweight at any age and adiposopathic markers differed by gender (Supplementary Table S7). There was some evidence that the associations between overweight at adult ages and inflammatory markers (CRP and IL-6) varied by gender, though no other adiposipathic markers showed consistent gender interaction. The main results were therefore presented for both genders combined. Gender-specific analyses of the associations between overweight at each age, fitted separately, and CRP and IL-6 indicated that the pattern of relationships was apparent for both genders but stronger in women; among whom independent associations between BMI in early adult life and adiposipathic markers were observed even after adjustment for BMI at 63 years (Supplementary Table S8).

In order to compare the fit of the different life course models, the sample was restricted further to participants who had BMI measurements at three specific age points, chosen to represent adolescence (15 years), early adulthood (36 years) and early old age (60–64 years; *n*=1280, 71.7%). Associations of overweight at each age with adiposopathic makers were almost identical, and interpretations unchanged, in the reduced sample (data not shown). Over the three ages, a total of 354 (27.7%) of participants were never overweight and 323 (25.5%) were only overweight at age 60–64 years. Among 537 (42.0%) study members who first became overweight at age 15 or 36 years (*n*=537), 474 (88.2%) subsequently remained overweight.

With adjustments for age and sex, the model where risk accumulated across all three life periods stood out as the best model to describe the relationship between life course overweight and the adipokines and inflammatory markers. For vWF, there was no evidence that the null model provided a poorer fit than the saturated model and so it was concluded that there was no effect of overweight at any age on vWF at age 60–64 years. Accumulation models including overweight at all three ages fitted the data as well as the saturated models for all other markers. The AIC for the model constraining the effect of overweight to be constant at all ages (‘strict’ accumulation model) had the lowest AIC for IL-6 and the model allowing the effect to vary across the three age points (‘relaxed’ accumulation) the lowest for leptin and CRP (Table 2). For the endothelial markers, E-selectin and t-PA, as well as for adiponectin, although the ‘relaxed’ accumulation model explained the data as well as the saturated model, the early old age sensitive period model had slightly smaller AICs. For consistency across types of markers, we thus investigated the relaxed accumulation model further for the adipokines and the inflammatory markers and the early old age model for the endothelial markers E-selectin and t-PA. We also investigated the early old age model for adiponectin and the strict accumulation model for adiponectin as the best fitting models according to the AIC.

Details of the selected life course models controlling for age, sex and potential confounders of smoking, childhood socioeconomic position and adult socioeconomic position are shown in Table 3. For adiponectin, leptin and CRP, after adjustment for overweight at the other two ages, the strengths of association with overweight increased with age. For IL-6, overweight at 60–64 years had the strongest association, followed by overweight at 15 years and then at 36 years. E-selectin and t-PA were, respectively, 16.2% (10.2, 22.5) and 34.3% (25.2, 44.0) higher for participants who were overweight compared with those not overweight at the age of 60–64 years. Adjustment for smoking status at age 60–64 years increased the strength of these associations slightly for adiponectin, CRP, E-selectin and IL-6, whereas adjustment for both childhood and adulthood socioeconomic measures reduced these associations slightly, but relationships remained strong after full adjustment.

All results were similar when alternative ages (20 rather than 15 years, 26 or 43 rather than 36 years, and 53 rather than 60–64 years) were used to test the fit of life course overweight models (data not shown). Exclusion of participants with CRP levels indicative of acute infection (⩾10) eliminated associations of overweight at age 2 with CRP and adiponectin but did not substantially alter other associations or life course modelling conclusions (data not shown). Excluding those with a self-report of CVD or stroke at age 53 or 60–64 years only altered associations slightly, both higher and lower, with interpretation of results not changed (data not shown). Results from models using multiple imputations were largely consistent with results from the complete case analyses. Associations of adiponectin with overweight at 2 years and e-selectin with 15 years were attenuated in the multiple imputation analysis, while associations of adiponectin and e-selectin with overweight at 20 years were apparent in the multiple imputation and not the complete case analysis (Supplementary Table S9).

## Discussion

In this cohort of British men and women followed prospectively from birth in 1946 until age 60–64 years, greater accumulation of overweight across adolescence and adulthood was associated with higher adverse levels of several adiposopathic markers, particularly adipokines and inflammatory markers, which are closely implicated in the development of the atherosclerotic process. A slightly different pattern was seen for the endothelial makers of E-selectin and t-PA, where overweight at 60–64 years was particularly important.

The finding that overweight status was associated with levels of adiponectin, leptin, CRP and IL-6 is consistent with many previous studies showing that excess body fat in adulthood is associated cross-sectionally with adverse levels of these adipokines^12, 13, 20^ and inflammatory markers.^15, 18^ However, the current analyses extend earlier reports by including a wider range of adiposopathic markers, including markers of endothelial dysfunction. We were able to show that, even though all of the outcomes measured for this study are a part of the same adiposopathic pathway, differences between overweight and non-overweight study members were much larger for leptin than the other markers. This could be explained by the fact that leptin is a very strong marker of total body fat status and a key regulator of energy intake and expenditure through appetite, metabolism and behaviour.^6^ Adiponectin is also directly released by adipose tissue, but is less directly related to body fat, potentially because of differences in how each adipokine is expressed in fat cells, structure and regulation. As the changes in inflammatory and endothelial marker concentrations could be a consequence of the changes in adipokines, it is logical that strengths of association would be weaker. The absence of a consistent association between overweight status and vWF is consistent with earlier reports showing that vWF is less consistently associated with body fatness than other endothelial function markers.^30, 31^

The current report is also important in showing that not only does being overweight at one period in time equate to more adverse levels of adipokines and inflammatory markers but also that continuing to stay overweight in adulthood will lead to increasingly adverse levels of these adiposopathic markers. A large number of randomised controlled trials have documented that weight loss in adulthood (generally >5%) can lead to lower levels of leptin,^17, 18^ CRP and IL-6 (refs 14,17) and higher levels of adiponectin.^14, 17, 18^ Randomised controlled trials that did not find an effect of weight loss tended to be of short duration (<8 weeks) or small sample sizes. In observational longitudinal studies, the Cardiovascular Health Study found that weight loss in elder adults over a 3-year period was associated with lower CRP, whereas weight gain was associated with higher CRP,^15^ and increases in waist circumference of Afro–Jamaican women over an average of 4.1 years was associated with lower adiponectin levels.^16^

Explanations for why the strength of associations between overweight status and adiposopathic markers increased with age are unknown. One possibility is that the estimates are actually the same across adulthood, but that associations for earlier adult ages appear weaker owing to BMI measurements being taken further away in time from when the adiposopathic markers were assessed. A more likely explanation is that study members who were overweight at one age were likely to be overweight at the next age (that is, tracking), with those overweight longer likely to have higher BMIs on average;^32^ and hence there would be larger differences in the adiposopathic markers between the overweight and non-overweight groups at later ages. Tracking of overweight over the life course is also why associations of overweight at earlier years with markers were reduced after adjustment for overweight at 60–64 years. Even with strong effects of overweight at older ages, estimates after adjustment for current overweight, and the results of the structured modelling approach, indicated that overweight at periods earlier than 60–64 years were important for adiposipathic marker levels at 60–64 years.

There was also evidence that differences in the adiposopathic markers at age 60–64 years for overweight compared with non-overweight widened over adulthood faster for some markers than others. This is the explanation for why the best fitting model chosen for each adiposopathic marker was generally some form of an accumulation model, but with heterogeneity in the type of accumulation. For example, the best fitting life course model chosen for IL-6 was the strict accumulation model because increases in associations were relatively small and consistent over time. For adiponectin and leptin, increases in associations by age increased stepwise from ages 15–36 to 60–64, resulting in a life course model chosen where the effect of overweight accumulates over time, but being overweight at older ages has more of an effect than being overweight earlier in adulthood.

For E-selectin and t-PA, effects of overweight appeared to be particularly strong for the cross-sectional measurement (60–64 years), but the possible influence of overweight at earlier periods should not be discounted. Similar to the inflammatory and endothelial markers, overweight during adolescence and adulthood was associated with the endothelial markers. That all associations were explained by overweight at 60–64 suggests that all relationships seen for overweight at earlier years could be due to tracking of body fatness. However, the finding that model fit for the sensitive period at 60–64 and the accumulation models were very similar suggests that overweight in adolescence and young adulthood are determinants of endothelial function in middle age, but that overweight in middle age is the dominant influence.

Important strengths of this study were the availability of data on a wide range of markers involved in the adiposopathic pathway and prospective objective measures of height and weight across the life course.^33^ We defined overweight as a dichotomous variable using a standard definition to facilitate the use of a modelling approach that is an improvement over traditional regression methods, in that we can attempt to disentangle how an exposure might be linked to an outcome by comparing the fit of the data of two or more hypothesised life course models; as compared with traditional regression that may miss an important information by only testing one model at a time.^28^ For example, as strengths of association between overweight and markers increased with age, and associations were attenuated by adjustment for overweight at 60–64 years, conclusions could be made that overweight at earlier ages did not matter. However, the method used in our paper shows that it is overweight at multiple ages over adolescence and adulthood accumulating to produce adipokine and inflammatory values in late middle age.

The life course modelling approach does have its limitations however. For most of the adiposipathic markers, two or more of the life course models explained the data as well as the saturated model making it difficult to determine a single best fitting model. We chose to interpret the model with the lowest AIC as the ‘best fitting’ life course model while also investigating models with similar fit. In reality, the processes linking excess body weight across the life course to the adiposopathic markers may not conform to any of the life course models specified here; if this were the case, it would be impossible to identify the relevant model from these analyses.^28^ Also, in order to maintain a reasonable number of participants in each trajectory group, and to reduce the potential multi-colinearity between multiple BMI measurements, we dichotomised BMI using the standard cut-off for overweight and only included three ages selected a priori to represent three different periods of the life course. Although BMI values at the three age points were correlated, the proportions of variance in BMI at 60–64 years explained were low, particularly in adolescence; directions of associations and variances were stable in multivariable models. Findings were robust to the use of alternative BMI cut-off values for overweight categories and all alternative combinations of ages, with variance and direction of estimates stable in multivariable models, suggesting the analysis was not biased by these analytical decisions. The limitations of BMI as a marker of body fatness are well known,^34^ particularly in children, but it is a widely used measure easily applied to clinical practise. As in any longitudinal study, attrition of the sample occurred, despite high response rates.^23^ An advantage of using the NSHD is the rich socioeconomic and health information that has been collected from birth, allowing us to confirm that results did not differ appreciably when missing data were imputed from current and prior data collections. The NSHD sample were all born in England, Scotland and Wales in March 1946, before the main immigration flows, and therefore findings cannot be extrapolated to the non-white British population, of whom only a small minority of the are currently 60 years or older.^22^

In conclusion, we provide evidence that the longer an individual is overweight during adolescence and adulthood, the more adverse their level of adipokines and inflammatory markers at 60–64 years. Given that children alive today are likely to spend more of their lives as overweight or obese than study members in this study, with 33.9% of children in England currently being overweight or obese by the age of 10–11 years,^35^ compared with only 10.2% for NSHD at the same age;^36^ the differences seen in these adiposopathic markers between overweight and normal weight individuals could be even larger when current generations reach retirement age. The results suggest that the prevention of excess adiposity early in the life course could be important for the prevention of CVD in adult life. In addition, many of these adiposopathic risk factors are related to type 2 diabetes risk as well as to CVD.^37^ Therefore, preventing the development of overweight and obesity in early adult life or earlier could have appreciable benefit for the prevention of type 2 diabetes as well as CVD.

## Figures and Tables

**Table 1:** Description of study members in the 1946 British Birth cohort with complete data on adiposopathic markers at age 60–64 years

	*Total*		*Men*		*Women*		P*-value sex difference*
	N	*Mean (s.d.)*	N	*Mean (s.d.)*	N	*Mean (s.d.)*	
Mean age (years)	1777	63.2 (1.1)	868	63.1 (1.2)	909	63.2 (1.1)	0.14
Mean adiponectin (ug ml^−1^)^a^	1784	2.5 (0.7)	873	2.2 (0.7)	911	2.8 (0.6)	<0.01
Mean leptin (ng ml^−1^)^a^	1784	2.5 (0.9)	873	2.0 (0.8)	911	3.0 (0.8)	<0.01
Mean E-selectin (ng ml^−1^)^a^	1784	3.6 (0.5)	873	3.6 (0.5)	911	3.5 (0.4)	<0.01
Mean t-PA (ng ml^−1^)^a^	1784	2.1 (0.6)	873	2.2 (0.6)	911	2.1 (0.6)	<0.01
Mean vWF (IU ml^−1^)^a^	1784	4.8 (0.5)	873	4.8 (0.5)	911	4.8 (0.5)	0.83
Mean CRP (mg l^−1^)^a^	1781	0.8 (0.9)	872	0.8 (0.9)	909	0.9 (0.9)	0.12
Mean IL-6 (pg ml^−1^)^a^	1784	0.7 (0.7)	873	0.7 (0.7)	911	0.7 (0.7)	0.05
							
*Overweight (obese, years),* n *(%)*^b^							
2	1397	35.2 (15.2)	692	33.8 (16.5)	705	36.5 (13.9)	0.3
4	1551	20.3 (5.5)	762	20.7 (6.7)	789	19.8 (4.4)	0.64
6	1461	10.5 (0.8)	719	9.7 (0.6)	742	11.3 (1.1)	0.32
7	1502	7.5 (0.8)	738	5.6 (0.3)	764	9.3 (0.8)	0.01
11	1508	8.0 (0.8)	741	6.6 (0.7)	767	9.3 (0.9)	0.06
15	1384	8.6 (0.6)	683	6.9 (0.4)	701	10.3 (0.7)	0.03
20	1464	11.7 (1.0)	701	13.1 (0.4)	763	10.4 (1.6)	0.1
26	1567	18.0 (1.8)	759	23.1 (2.0)	808	13.2 (1.6)	<0.01
36	1624	31.2 (4.3)	792	40.9 (3.9)	832	21.9 (4.6)	<0.01
43	1677	44.3 (9.6)	819	52.8 (8.6)	310	36.1 (10.6)	<0.01
53	1701	65 (20.9)	823	71.2 (19.7)	878	59.3 (22.0)	<0.01
60–64	1781	69.5 (27.5)	871	73.9 (26.6)	910	65.3 (28.4)	<0.01
							
CVD (%)	1711	8.8	828	11.5	883	6.3	<0.01
Current smoker (%)	1634	10.8	796	10.3	838	11.3	0.5
Ex-smoker (%)	1634	41.1	796	48.1	838	34.4	<0.01
Manual CSEP (%)	1699	53	835	53.5	864	52.6	0.68
Manual ASEP (%)	1774	27.4	866	32	908	23	<0.01

Abbreviations: ASEP, adult socioeconomic position; BMI, body mass index; CRP, C-reactive protein; CSEP, childhood socioeconomic position; CVD, cardiovascular disease; IL-6, interleukin-6; t-PA, tissue plasminogen activator; vWF, von Willebrand factor.

Geometric mean.

Overweight=BMI⩾25 kg m^−2^; obese=BMI⩾30 kg m^−2^.

**Table 2:** Results of *P*-values^a^ for partial F-tests, and AIC, comparing each represented life course models to a saturated model^b^ presented for each adiposopathic marker separately (*n*=1280)

	*Adipokines*						*Endothelial markers*				*Inflammatory markers*			
	*Adiponectin*		*Leptin*		*E-selectin*		*t-PA*		*vWF*		*CRP*		*IL-6*	
	P*-value*	*AIC*	P*-value*	*AIC*	P*-value*	*AIC*	P*-value*	*AIC*	P*-value*	*AIC*	P*-value*	*AIC*	P*-value*	*AIC*
Saturated model	—	2493.34	—	2454.81	—	1517.64	—	2230.56	—	2045.42	—	3221.56	—	2629.13
No effect	0.00	2541.03	0.00	2974.35	0.00	1542.49	0.00	2289.44	*0.74*	2035.95	0.00	3269.2	0.00	2665.52

*Sensitive period (years)* c														
Adolescence (15)	0.00	2542.00	0.00	2945.47	0.00	1540.75	0.00	2288.06	**0.66**	—	0.00	3264.84	0.00	2655.79
Adulthood (36)	0.00	2525.86	0.00	2819.77	0.00	1535.12	0.00	2273.38	**0.90**	—	0.00	3246.25	0.00	2645.53
Early old age (60–64)	**0.59**	2486.05	0.00	2491.99	*0.24*	1513.67	*0.42*	2224.53	**0.90**	—	0.05	3222.08	0.00	2636.04

*Accumulation* d														
Adolescence+adulthood	0.00	2529.48	0.00	2823.73	0.00	1533.73	0.00	2274.18	**0.87**	—	0.00	3245.62	0.00	2639.94
Adulthood+early old age	0.07	2493.25	0.00	2529.55	**0.07**	1517.52	0.01	2235.21	**0.98**	—	0.18	3218.56	0.07	2628.77
All three, relaxed^e^	*0.66*	2487.77	*0.11*	2454.32	**0.21**	1515.52	**0.40**	2226.5	**0.88**	—	*0.34*	3218.08	**0.19**	2627.35
All three, strict^f^	0.00	2500.89	0.00	2564.25	**0.06**	1517.77	0.00	2240.31	**0.74**	—	**0.12**	3219.85	*0.30*	2624.45

Abbreviations: AIC, Akaike Information Criteria; CRP C-reactive protein; IL-6 interleukin-6; t-PA tissue plasminogen activator; vWF von Willebrand factor.

a A higher *P*-value for the life course model equals a better model fit.

b The ‘saturated model’ is most complicated model that contains overweight at all three ages, all two-way interactions and the three-way interaction.

c A ‘sensitive period’ model refers to testing whether being overweight at one age only (15, 36 or 60–64) is a better predictor of the adiposopathic marker at age 60–64 years than the saturated model.

d The ‘accumulation’ models indicate that being overweight at more than one age contributes to differences in adiposopathic markers at 60–64 years.

e A ‘relaxed’ accumulation model indicates that associations of overweight at each age can have different strengths.

f A ‘strict’ accumulation model indicates strengths of association are equal for all the three ages. Bold values indicate *P*-value>0.05.

**Table 3:** Percentage increase (95% CI) of each adiposopathic marker for the selected life course model (*n*=1280)

	*Age and sex only*	*+Smoking status*	*+Childhood SEP*	*+Adulthood SEP*	*Full model*
*Accumulation model, relaxed* a					
Adiponectin (years)					
15	2.2 (−10.3, 16.4)	0.7 (−11.6, 14.7)	2.2 (−10.2, 16.4)	2.3 (−10.3, 16.5)	1.1 (−11.2, 15.2)
36	−6.3 (−13.9, 2.0)	−4.7 (−12.7, 4.0)	−6.0 (−13.7, 2.3)	−6.6 (−14.3, 1.8)	−4.6 (−12.7, 4.2)
63	−24.3 (−30.4, −17.7)	−26.1 (−32.2, −19.4)	−23.8 (−30.0, −17.2)	−24.2 (−30.3, −17.6)	−25.8 (−32.0, −19.1)
					
Leptin (years)					
15	15.4 (1.5, 31.2)	17.8 (3.3, 34.4)	15.4 (1.5, 31.2)	17.0 (2.9, 33.1)	19.3 (4.6, 36.0)
36	26.6 (16.4, 37.6)	23.1 (12.6, 34.4)	25.8 (15.7, 36.8)	26.0 (15.8, 37.1)	21.7 (11.3, 33.0)
63	135.9 (117.2, 156.2)	135.0 (115.4, 156.4)	133.4 (114.8, 153.6)	135.6 (117.0, 155.9)	132.6 (113.1, 153.9)
					
CRP (years)					
15	10.1 (−7.5, 31.0)	10.3 (−7.7, 31.8)	10.2 (−7.4, 31.1)	12.6 (−5.4, 33.9)	11.9 (−6.3, 33.7)
36	14.4 (2.1, 28.1)	13.9 (1.0, 28.4)	13.1 (0.9, 26.6)	13.9 (1.7, 27.7)	13.1 (0.3, 27.6)
63	37.0 (22.5, 53.1)	39.1 (23.6, 56.5)	34.3 (20.1, 50.3)	35.5 (21.2, 51.4)	36.1 (20.9, 53.3)
					
Interleukin-6 (years)^b^					
15	16.4 (1.5, 33.6)	19.1 (3.7, 36.8)	16.5 (1.6, 33.7)	18.3 (3.1, 35.7)	20.9 (5.3, 38.9)
36	11.1 (1.6, 21.5)	8.9 (−0.8, 19.6)	10.1 (0.7, 20.5)	10.6 (1.1, 21.1)	8.5 (−1.2, 19.1)
63	20.1 (10.0, 31.2)	20.7 (10.1, 32.3)	18.5 (8.4, 29.5)	19.5 (9.4, 30.5)	19.5 (9.0, 31.1)
					
*Early old age sensitive period model (60–64 years only)* c					
Adiponectin	−30.1 (−37.8, −22.3)	−32.0 (−39.9, −24.1)	−29.5 (−37.3, −21.7)	−29.8 (−37.6, −22.0)	−29.8 (−37.8, −21.8)
E-selectin	16.2 (10.2, 22.5)	17.5 (11.1, 24.2)	15.4 (9.4, 21.8)	16.1 (10.0, 22.4)	16.6 (10.1, 23.4)
Tissue plasminogen activator	34.3 (25.2, 44.0)	31.3 (21.9, 41.3)	33.9 (24.7, 43.7)	34.2 (25.1, 44.0)	30.9 (21.4, 41.1)

Abbreviations: CI, confidence interval; CRP, C-reactive protein; IL-6, interleukin-6; SEP, socioeconomic position.

a Overweight compared with non-overweight at 15, 36 or 60–64 years, adjusted for overweight status for the other 2 years.

b The ‘strict’ accumulation model was chosen as the best fitting life course model for IL-6, but *P*-values were similar to the ‘relaxed’ accumulation model, so we have shown the latter model here to compare coefficients for overweight at each age with the other inflammatory marker.

c Overweight compared with non-overweight at age 60–64 years.

## References

[bib1] World Health OrganizationGlobal Health Risks: Mortality and Burden Of Disease Attributable To Selected Major Risks. WHO Press: Geneva, Switzerland, 2009.

[bib2] Lim SS, Vos T, Flaxman AD, Danaei G, Shibuya K, Adair-Rohani H et al. A comparative risk assessment of burden of disease and injury attributable to 67 risk factors and risk factor clusters in 21 regions, 1990-2010: a systematic analysis for the Global Burden of Disease Study 2010. Lancet 2012; 380: 2224–2260. 23245609 10.1016/S0140-6736(12)61766-8 PMC4156511

[bib3] Institute for Health Metrics and EvaluationThe Global Burden of Disease: Generating Evidence, Guiding Policy. IHME: Seattle, Washington, USA, 2013.

[bib4] Ntaios G, Gatselis NK, Makaritsis K, Dalekos GN. Adipokines as mediators of endothelial function and atherosclerosis. Atherosclerosis 2013; 227: 216–221. 23332774 10.1016/j.atherosclerosis.2012.12.029

[bib5] Adamczak M, Wiecek A. The adipose tissue as an endocrine organ. Semin Nephrol 2013; 33: 2–13. 23374889 10.1016/j.semnephrol.2012.12.008

[bib6] Galic S, Oakhill JS, Steinberg GR. Adipose tissue as an endocrine organ. Mol Cell Endocrinol 2010; 316: 129–139. 19723556 10.1016/j.mce.2009.08.018

[bib7] Van d V, Pauwels B, Boydens C, Decaluwe K. Adipocytokines in relation to cardiovascular disease. Metabolism 2013; 62: 1513–1521. 23866981 10.1016/j.metabol.2013.06.004

[bib8] James WP. Obesity-a modern pandemic: the burden of disease. Endocrinol Nutr 2013; 60: 3–6. 24490215 10.1016/s1575-0922(13)70015-9

[bib9] Baker JL, Olsen LW, Sorensen TI. Childhood body-mass index and the risk of coronary heart disease in adulthood. N Engl J Med 2007; 357: 2329–2337. 18057335 10.1056/NEJMoa072515 PMC3062903

[bib10] Owen CG, Whincup PH, Orfei L, Chou QA, Rudnicka AR, Wathern AK et al. Is body mass index before middle age related to coronary heart disease risk in later life? Evidence from observational studies. Int J Obes (Lond) 2009; 33: 866–877. 19506565 10.1038/ijo.2009.102 PMC2726133

[bib11] Tirosh A, Shai I, Afek A, Dubnov-Raz G, Ayalon N, Gordon B et al. Adolescent BMI trajectory and risk of diabetes versus coronary disease. N Engl J Med 2011; 364: 1315–1325. 21470009 10.1056/NEJMoa1006992 PMC4939259

[bib12] Marques-Vidal P, Bochud M, Paccaud F, Mooser V, Waeber G, Vollenweider P. Distribution of plasma levels of adiponectin and leptin in an adult Caucasian population. Clin Endocrinol (Oxf) 2010; 72: 38–46. 19473178 10.1111/j.1365-2265.2009.03628.x

[bib13] Rasmussen-Torvik LJ, Wassel CL, Ding J, Carr J, Cushman M, Jenny N et al. Associations of body mass index and insulin resistance with leptin, adiponectin, and the leptin-to-adiponectin ratio across ethnic groups: the Multi-Ethnic Study of Atherosclerosis (MESA). Ann Epidemiol 2012; 22: 705–709. 22929534 10.1016/j.annepidem.2012.07.011 PMC3459265

[bib14] Ambeba EJ, Styn MA, Kuller LH, Brooks MM, Evans RW, Burke LE. Longitudinal effects of weight loss and regain on cytokine concentration of obese adults. Metabolism 2013; 62: 1218–1222. 23725640 10.1016/j.metabol.2013.04.004 PMC4266426

[bib15] Barzilay JI, Forsberg C, Heckbert SR, Cushman M, Newman AB. The association of markers of inflammation with weight change in older adults: the Cardiovascular Health Study. Int J Obes (Lond) 2006; 30: 1362–1367. 16534520 10.1038/sj.ijo.0803306

[bib16] Boyne MS, Bennett NR, Cooper RS, Royal-Thomas TY, Bennett FI, Luke A et al. Sex-differences in adiponectin levels and body fat distribution: longitudinal observations in Afro-Jamaicans. Diabetes Res Clin Pract 2010; 90: e33–e36. 20828849 10.1016/j.diabres.2010.08.008 PMC2953571

[bib17] Forsythe LK, Wallace JM, Livingstone MB. Obesity and inflammation: the effects of weight loss. Nutr Res Rev 2008; 21: 117–133. 19087366 10.1017/S0954422408138732

[bib18] Heinonen MV, Laaksonen DE, Karhu T, Karhunen L, Laitinen T, Kainulainen S et al. Effect of diet-induced weight loss on plasma apelin and cytokine levels in individuals with the metabolic syndrome. Nutr Metab Cardiovasc Dis 2009; 19: 626–633. 19278844 10.1016/j.numecd.2008.12.008

[bib19] Nazmi A, Gonzalez DC, Oliveira IO, Horta BL, Gigante DP, Victora CG. Life course weight gain and C-reactive protein levels in young adults: findings from a Brazilian birth cohort. Am J Hum Biol 2009; 21: 192–199. 19107921 10.1002/ajhb.20852

[bib20] Rasmussen-Torvik LJ, Pankow JS, Jacobs DRJr, Steinberger J, Moran A, Sinaiko AR. Development of associations among central adiposity, adiponectin and insulin sensitivity from adolescence to young adulthood. Diabet Med 2012; 29: 1153–1158. 22672197 10.1111/j.1464-5491.2012.03726.x PMC3418404

[bib21] Tzoulaki I, Jarvelin MR, Hartikainen AL, Leinonen M, Pouta A, Paldanius M et al. Size at birth, weight gain over the life course, and low-grade inflammation in young adulthood: northern Finland 1966 Birth Cohort study. Eur Heart J 2008; 29: 1049–1056. 18403494 10.1093/eurheartj/ehn105

[bib22] Kuh D, Pierce M, Adams J, Deanfield J, Ekelund U, Friberg P et al. Cohort profile: updating the cohort profile for the MRC National Survey of Health and Development: a new clinic-based data collection for ageing research. Int J Epidemiol 2011; 40: e1–e9. 21345808 10.1093/ije/dyq231 PMC3043283

[bib23] Stafford M, Black S, Shah I, Hardy R, Pierce M, Richards M et al. Using a birth cohort to study ageing: representativeness and response rates in the National Survey of Health and Development. Eur J Ageing 2013; 10: 145–157. 23637643 10.1007/s10433-013-0258-8 PMC3637651

[bib24] Cole TJ, Bellizzi MC, Flegal KM, Dietz WH. Establishing a standard definition for child overweight and obesity worldwide: international survey. BMJ 2000; 320: 1240–1243. 10797032 10.1136/bmj.320.7244.1240 PMC27365

[bib25] Galobardes B, Shaw M, Lawlor DA, Lynch JW, Davey SG. Indicators of socioeconomic position (part 2). J Epidemiol Community Health 2006; 60: 95–101. 16415256 10.1136/jech.2004.028092 PMC2566160

[bib26] Pierce MB, Silverwood RJ, Nitsch D, Adams JE, Stephen AM, Nip W et al. Clinical disorders in a post war British cohort reaching retirement: evidence from the First National Birth Cohort study. PLoS One 2012; 7: e44857. 23028647 10.1371/journal.pone.0044857 PMC3447001

[bib27] Cole TJ. Sympercents: symmetric percentage differences on the 100 log€ scale simplify the presentation of log transformed data. Stat Med 2000; 19: 3109–3125. 11113946 10.1002/1097-0258(20001130)19:22<3109::aid-sim558>3.0.co;2-f

[bib28] Mishra G, Nitsch D, Black S, De SB, Kuh D, Hardy R. A structured approach to modelling the effects of binary exposure variables over the life course. Int J Epidemiol 2009; 38: 528–537. 19028777 10.1093/ije/dyn229 PMC2663717

[bib29] Sattar N, Wannamethee SG, Forouhi NG. Novel biochemical risk factors for type 2 diabetes: pathogenic insights or prediction possibilities? Diabetologia 2008; 51: 926–940. 18392804 10.1007/s00125-008-0954-7

[bib30] Wannamethee SG, Tchernova J, Whincup P, Lowe GD, Kelly A, Rumley A et al. Plasma leptin: associations with metabolic, inflammatory and haemostatic risk factors for cardiovascular disease. Atherosclerosis 2007; 191: 418–426. 16712853 10.1016/j.atherosclerosis.2006.04.012

[bib31] Park MH, Sovio U, Viner RM, Hardy RJ, Kinra S. Overweight in childhood, adolescence and adulthood and cardiovascular risk in later life: pooled analysis of three british birth cohorts. PLoS One 2013; 8: e70684. 23894679 10.1371/journal.pone.0070684 PMC3722162

[bib32] Hattori A, Sturm R. The obesity epidemic and changes in self-report biases in BMI. Obesity (Silver Spring) 2013; 21: 856–860. 23712990 10.1002/oby.20313 PMC5800501

[bib33] Huxley R, Mendis S, Zheleznyakov E, Reddy S, Chan J. Body mass index, waist circumference and waist:hip ratio as predictors of cardiovascular risk--a review of the literature. Eur J Clin Nutr 2010; 64: 16–22. 19654593 10.1038/ejcn.2009.68

[bib34] Cole TJ. A method for assessing age-standardized weight-for-height in children seen cross-sectionally. Ann Hum Biol 1979; 6: 249–268. 496386 10.1080/03014467900007252

[bib35] The Health and Social Care Information Centre. National Child Measurement Programme - England, 2011-2012 school year [NS], 12 December 2012. Report no. 1.

[bib36] Cooper R, Hardy R, Kuh D. Is adiposity across life associated with subsequent hysterectomy risk? Findings from the 1946 British birth cohort study. BJOG 2008; 115: 184–192. 18081600 10.1111/j.1471-0528.2007.01569.x

[bib37] Goldberg RB. Cytokine and cytokine-like inflammation markers, endothelial dysfunction, and imbalanced coagulation in development of diabetes and its complications. J Clin Endocrinol Metab 2009; 94: 3171–3182. 19509100 10.1210/jc.2008-2534

