# Supplementary material for: Overweight across the life course and adipokines, inflammatory and endothelial markers at age 60–64 years: evidence from the 1946 birth cohort
Source: Int J Obes (Lond). 2015 Jun 9;39(6):1010–8. doi: 10.1038/ijo.2015.19 (PMC4433551; doi:10.1038/ijo.2015.19)
Supplement: Supplementary Tables [file ijo201519x1.docx]

**Table S1.** Methods and interassay coefficients (CV) of variation for risk factors assessed from blood samples

| Risk factor | Units | Assay/Method | CV (%) |
| --- | --- | --- | --- |
| **Novel risk factors** |  |  |  |
| E-selectin | ng/ml | High sensitivity ELISA | <10.0% |
| C-reactive protein (CRP) | mg/l | Particle-enhanced immunoturbidimetric assay | 4.3% at 3.4 mg/L 1.8% at 11.9 mg/L |
| Interleukin-6 (IL6) | pg/ml | Enzyme-linked immunosorbent assay (ELISA) | 6.5% |
| Tissue plasminogen activator (tPA) | ng/ml | ELISA | 6.6% |
| Leptin | ng/ml | In-house radioimmunoassay validated against commercially available assays | <10.0% |
| Adiponectin | *μ*g/ml | ELISA | <7.5% |

* Any results that fell below the lower detection limit of the assay were assigned a notional value, obtained by dividing the lower detection

limit by the square root of 2.

**Table S2.** Correlations of body mass index between ages of examination

|  | 2 yrs | 4 yrs | 6 yrs | 7 yrs | 11 yrs | 15 yrs | 20 yrs | 26 yrs | 36 yrs | 43 yrs | 53 yrs | 60-64 yrs |
| --- | --- | --- | --- | --- | --- | --- | --- | --- | --- | --- | --- | --- |
| 2 yrs | - |  |  |  |  |  |  |  |  |  |  |  |
| 4 yrs | 0.24 | - |  |  |  |  |  |  |  |  |  |  |
| 6 yrs | 0.30 | 0.42 | - |  |  |  |  |  |  |  |  |  |
| 7 yrs | 0.21 | 0.42 | 0.68 | - |  |  |  |  |  |  |  |  |
| 11 yrs | 0.14 | 0.30 | 0.54 | 0.64 | - |  |  |  |  |  |  |  |
| 15 yrs | 0.10 | 0.28 | 0.50 | 0.57 | 0.78 | - |  |  |  |  |  |  |
| 20 yrs | 0.10 | 0.25 | 0.39 | 0.45 | 0.59 | 0.62 | - |  |  |  |  |  |
| 26 yrs | 0.11 | 0.20 | 0.29 | 0.32 | 0.49 | 0.53 | 0.78 | - |  |  |  |  |
| 36 yrs | 0.13 | 0.24 | 0.30 | 0.32 | 0.49 | **0.50** | 0.65 | 0.76 | - |  |  |  |
| 43 yrs | 0.09 | 0.19 | 0.26 | 0.28 | 0.43 | 0.47 | 0.59 | 0.70 | 0.86 | - |  |  |
| 53 yrs | 0.06 | 0.15 | 0.21 | 0.22 | 0.39 | 0.43 | 0.51 | 0.63 | 0.79 | 0.86 | - |  |
| 60-64 yrs | 0.06 | 0.15 | 0.22 | 0.23 | 0.37 | 0.41 | 0.47 | 0.56 | **0.73** | 0.79 | 0.86 | - |

**Table S3.** Percentage increase (95% CI) of each adiposopathic marker (P-values) for each adiposopathic marker for overweight (BMI>=25 kg/m^2^) vs not overweight (BMI<25), adjusted for sex- and age at examination and fitted separately for each age.

|  | **Adipokines** | | | | **Inflammatory markers** | | | | **Endothelial markers** | | | | | |
| --- | --- | --- | --- | --- | --- | --- | --- | --- | --- | --- | --- | --- | --- | --- |
| **Overweight** | Adiponectin | | Leptin | | CRP | | IL-6 | | E-selectin | | t-PA | | vWF | |
|  | PI  (95% CI) | *p* | PI  (95% CI) | *p* | PI  (95% CI) | *p* | PI  (95% CI) | *p* | PI  (95% CI) | *p* | PI  (95% CI) | *p* | PI  (95% CI) | *p* |
| 2 years | 7.9  (0.3, 16.2) | *.04* | -5.6  (-13.2, 2.6) | *.18* | **12.7**  **(2.5, 24.0)** | ***.01*** | 5.1  (-2.7, 13.5) | *.21* | 2.7  (-2.4, 8.0) | *.30* | 3.1  (-3.7, 10.3) | *.38* | 3.8  (-2.1, 10.1) | *.21* |
| 4 years | -1.0  (-8.8, 7.5) | *.82* | -7.0  (-15.4, 2.4) | *.14* | -1.8  (-11.9, 9.5) | *.75* | 1.2  (-7.2, 10.4) | *.79* | 0.1  (-5.3, 5.9) | *.97* | 0.1  (-7.2, 7.9) | *.98* | -4.6  (-10.8, 2.0) | *.17* |
| 6 years | -2.8  (-12.9, 8.4) | *.61* | -3.9  (-15.5, 9.3) | *.55* | -2.0  (-15.2, 13.2) | *.78* | 2.7  (-8.6, 15.4) | *.65* | -2.8  (-9.9, 4.9) | *.47* | -1.7  (-11.2, 8.8) | *.74* | **12.2**  **(2.5, 22.8)** | ***.01*** |
| 7 years | 5.0  (-7.5, 19.2) | *.45* | 0.8  (-13.2, 17.1) | *.92* | -1.2  (-16.3, 16.6) | *.89* | -8.7  (-20.0, 4.2) | *.18* | 1.3  (-7.1, 10.4) | *.78* | -4.3  (-14.9, 7.6) | *.47* | -1.9  (-11.5, 8.8) | *.72* |
| 11 years | **-13.1**  **(-23.1, -1.7)** | ***.03*** | **50.0**  **(30.3, 72.6)** | ***<.01*** | 8.7  (-7.7, 28.0) | *.32* | **15.2**  **(1.2, 31.1)** | ***.03*** | 3.4  (-4.9, 12.5) | *.43* | 10.5  (-1.3, 23.7) | *.08* | 4.0  (-6.1, 15.1) | *.46* |
| 15 years | -7.2  (-18.0, 5.1) | *.24* | **50.3**  **(30.1, 73.6)** | ***<.01*** | **27.5**  **(8.2, 50.2)** | ***<.01*** | **29.1**  **(13.3, 47.1)** | ***<.01*** | **9.9**  **(1.0, 19.5)** | ***.03*** | **13.2**  **(1.1, 26.8)** | ***.03*** | 3.4  (-6.7, 14.6) | *.52* |
| 20 years | -9.5  (-18.5, 0.6) | *.07* | **48.1**  **(31.0, 67.5)** | ***<.01*** | **28.7**  **(12.1, 47.8)** | ***<.01*** | **18.9**  **(6.4, 32.9)** | ***<.01*** | **7.5**  **(0.2, 15.4)** | ***<.05*** | 5.0  (-4.7, 15.7) | *.32* | 4.9  (-3.9, 14.4) | *.29* |
| 26 years | **-11.4**  **(-18.7, -3.5)** | ***.01*** | **75.1**  **(58.9, 92.9)** | ***<.01*** | **28.3**  **(14.6, 43.5)** | ***<.01*** | **21.7**  **(11.4, 33.0)** | ***<.01*** | **7.1**  **(1.0, 13.6)** | ***.02*** | **10.3**  **(2.1, 19.3)** | ***.01*** | 6.6  (-0.7, 14.3) | *.08* |
| 36 years | **-16.3**  **(-22.0, -10.3)** | ***<.01*** | **73.6**  **(60.4, 87.9)** | ***<.01*** | **31.7**  **(20.0, 44.5)** | ***<.01*** | **20.8**  **(12.2, 30.1)** | ***<.01*** | **9.6**  **(4.4, 15.0)** | ***<.01*** | **17.2**  **(9.8, 25.0)** | ***<.01*** | 3.3  (-2.5, 9.4) | *.27* |
| 43 years | **-18.0**  **(-23.1, -12.7)** | ***<.01*** | **77.3**  **(65.3, 90.1)** | ***<.01*** | **40.5**  **(29.2, 52.7)** | ***<.01*** | **28.3**  **(20.1, 37.1)** | ***<.01*** | **9.7**  **(5.0, 14.6)** | ***<.01*** | **15.6**  **(9.0, 22.5)** | ***<.01*** | 3.8  (-1.6, 9.4) | *.17* |
| 53 years | **-26.8**  **(-31.4, -21.9)** | ***<.01*** | **101.9**  **(88.2, 116.5)** | ***<.01*** | **41.8**  **(30.2, 54.5)** | ***<.01*** | **32.4**  **(23.6, 41.8)** | ***<.01*** | **10.0**  **(5.2, 15.1)** | ***<.01*** | **25.8**  **(18.5, 33.7)** | ***<.01*** | 0.6  (-4.7, 6.1) | *.84* |
| 63 years | **-27.5**  **(-32.1, -22.7)** | ***<.01*** | **146.6**  **(130.8, 163.5)** | ***<.01*** | **45.4**  **(33.4, 58.6)** | ***<.01*** | **29.8**  **(21.1, 39.2)** | ***<.01*** | **15.8**  **(10.6, 21.2)** | ***<.01*** | **33.7**  **(25.9, 42.0)** | ***<.01*** | 5.1  (-0.6, 11.0) | *.07* |

**Table S4.** Percentage increase (95% CI) of each adiposopathic marker for a 1-SD increase in BMI (kg/m^2^), fitted separately for each age.

|  | **Adipokines** | | **Inflammatory markers** | | **Endothelial markers** | | |
| --- | --- | --- | --- | --- | --- | --- | --- |
|  |  | |  | |  | | |
| **Overweight** | Adiponectin | Leptin | CRP | IL-6 | E-selectin | t-PA | vWF |
| 2 years | 1.8 (-1.7, 5.4) | -3.2 (-7.0, 0.8) | **6.8 (2.0, 11.8)** | 2.7 (-1.0, 6.6) | 1.4 (-1.0, 3.9) | 1.4 (-1.8, 4.8) | 0.7 (-2.1, 3.6) |
| 4 years | 1.2 (-3.5, 6.2) | -3.8 (-9.1, 1.7) | 3.1 (-3.2, 9.9) | 2.5 (-2.6, 7.8) | 1.0 (-2.3, 4.3) | 1.9 (-2.5, 6.5) | -0.8 (-4.7, 3.1) |
| 6 years | 2.8 (-3.2, 9.2) | -3.9 (-10.5, 3.2) | -1.8 (-9.3, 6.4)* | 2.8 (-3.6, 9.6) | -1.6 (-5.6, 2.6) | -2.3 (-7.6, 3.3) | 2.0 (-2.9, 7.3)* |
| 7 years | 4.8 (-1.0, 10.9) | -3.3 (-9.5, 3.4) | -1.7 (-8.7, 5.8) | 2.0 (-3.9, 8.2) | -1.0 (-4.7, 2.9) | -2.6 (-7.6, 2.6) | -0.8 (-5.3, 3.8) |
| 11 years | -1.5 (-4.9, 2.1)* | **12.2 (7.8, 16.8)** | 2.5 (-2.2, 7.4) | **6.3 (2.5, 10.3)** | -0.1 (-2.5, 2.3) | 1.7 (-1.6, 5.0) | 0.7 (-2.2, 3.7) |
| 15 years | -2.1 (-5.3, 1.1) | **11.6 (7.5, 15.8)** | **5.3 (0.9, 9.9)** | **7.4 (3.9, 11.1)** | 2.2 (0.0, 4.4) | 2.2 (-0.8, 5.2) | 0.9 (-1.8, 3.6) |
| 20 years | -1.7 (-4.7, 1.4) | **13.1 (9.1, 17.3)** | **7.5 (3.3, 12.0)** | **7.0 (3.6, 10.5)** | 1.4 (-0.7, 3.6) | -0.5 (-3.3, 2.4) | 2.4 (-0.2, 5.1) |
| 26 years | **-3.1 (-5.7, -0.4)** | **20.9 (17.3, 24.7)** | **9.2 (5.5, 13.2)** | **9.1 (6.1, 12.1)** | **3.5 (1.6, 5.5)** | **3.1 (0.6, 5.6)** | **2.6 (0.4, 4.9)*** |
| 36 years | **-5.6 (-7.8, -3.4)** | **25.0 (21.8, 28.3)** | **11.9 (8.5, 15.4)** | **9.1 (6.5, 11.8)** | **3.1 (1.4, 4.8)** | **4.6 (2.4, 6.9)** | 1.7 (-0.2, 3.7) |
| 43 years | **-6.2 (-8.0, -4.3)** | **24.5 (21.9, 27.1)** | **11.3 (8.5, 14.3)** | **8.8 (6.6, 11.1)** | **2.8 (1.5, 4.2)** | **4.2 (2.3, 6.1)** | **2.5 (0.8, 4.1)*** |
| 53 years | **-7.3 (-8.8, -5.8)** | **25.3 (23.3, 27.4)** | **10.6 (8.3, 13.0)** | **8.7 (6.8, 10.6)** | **3.7 (2.6, 4.9)** | **5.7 (4.1, 7.3)** | **1.7 (0.4, 3.1)*** |
| 63 years | **-7.8 (-9.2, -6.4)** | **31.2 (29.4, 32.9)** | **12.6 (10.4, 14.9)** | **9.2 (7.5, 11.0)** | **4.8 (3.8, 6.0)** | **7.7 (6.2, 9.2)** | **2.5 (1.2, 3.8)*** |

**Table S5.** Percentage increase (95% CI) of each adiposopathic marker for overweight (BMI>=27.5 kg/m^2^) vs not overweight (BMI<27.5), fitted separately for each age.

|  | **Adipokines** | | **Inflammatory markers** | | **Endothelial markers** | | |
| --- | --- | --- | --- | --- | --- | --- | --- |
|  |  | |  | |  | | |
| **Overweight** | Adiponectin | Leptin | CRP | IL-6 | E-selectin | t-PA | vWF |
|  |  |  |  |  |  |  |  |
| 2 years | 1.8 (-6.7, 11.0) | -5.8 (-14.6, 4.0) | **13.3 (1.2, 26.9)** | 5.9 (-3.3, 16.0) | 5.8 (-0.4, 12.3) | 3.6 (-4.4, 12.3) | 1.6 (-5.3, 9.0) |
| 4 years | -2.1 (-11.8, 8.7) | -4.3 (-15.3, 8.1) | 1.3 (-11.8, 16.4) | -1.3 (-11.6, 10.3) | 2.0 (-5.0, 9.5) | 2.3 (-7.0, 12.6) | 1.2 (-7.1, 10.3) |
| 6 years | -18.2 (-33.7, 0.9) | 7.7 (-15.9, 38.0) | -18.3 (-38.1, 7.8) | -2.5 (-22.0, 21.9) | 10.6 (-4.3, 27.9) | 5.8 (-12.9, 28.6) | -5.1 (-20.3, 13.0) |
| 7 years | 17.3 (-10.1, 53.1) | 28.1 (-6.3, 75.2) | -16.9 (-41.3, 17.5) | -13.3 (-34.3, 14.3) | -0.5 (-17.0, 19.2) | -8.4 (-28.3, 17.2) | 6.7 (-14.1, 32.6) |
| 11 years | -15.1 (-31.7, 5.5) | **68.4 (31.1, 116.3)** | 16.6 (-12.7, 55.7) | 17.4 (-6.7, 47.7) | 11.8 (-3.7, 29.8) | 18.5 (-3.0, 44.6) | -1.1 (-17.4, 18.5) |
| 15 years | **-24.7 (-41.7, -2.9)** | **106.1 (53.2, 177.2)** | 33.3 (-4.9, 86.8) | **39.5 (6.6, 82.5)** | 17.7 (-1.0, 39.9) | 19.9 (-5.0, 51.3) | 14.2 (-7.5, 40.9) |
| 20 years | **-21.8 (-36.3, -4.1)** | **91.1 (50.5, 142.6)** | 23.1 (-6.2, 61.4) | 11.9 (-9.8, 38.9) | 8.6 (-5.3, 24.7) | -9.8 (-25.2, 8.9) | 9.3 (-7.7, 29.4) |
| 26 years | -9.6 (-21.9, 4.6) | **123.3 (89.2, 163.5)** | **39.5 (15.2, 69.0)** | **35.0 (16.2, 56.9)** | **14.2 (3.4, 26.1)** | **15.1 (0.9, 31.2)** | **15.6 (2.6, 30.1)** |
| 36 years | **-13.7 (-21.8, -4.9)** | **107.4 (85.7, 131.7)** | **31.7 (15.7, 50.0)** | **26.7 (14.3, 40.4)** | **10.5 (3.3, 18.2)** | 6.6 (-2.7, 16.7) | **11.0 (2.4, 20.3)** |
| 43 years | **-17.7 (-23.7, -11.3)** | **102.8 (86.7, 120.3)** | **40.5 (27.1, 55.2)** | **32.8 (22.8, 43.8)** | **10.8 (5.2, 16.7)** | **13.3 (5.7, 21.4)** | **10.7 (4.0, 17.9)** |
| 53 years | **-22.3 (-27.1, -17.3)** | **111.1 (97.6, 125.6)** | **36.4 (25.5, 48.3)** | **26.8 (18.6, 35.5)** | **15.8 (10.8, 20.9)** | **21.1 (14.2, 28.4)** | **6.1 (0.8, 11.8)** |
| 63 years | **-24.7 (-29.1, -20.0)** | **145.1 (131.0, 160.0)** | **49.4 (38.0, 61.8)** | **31.3 (23.2, 40.0)** | **19.1 (14.3, 24.2)** | **28.8 (21.8, 36.2)** | **6.6 (1.4, 12.2)** |

**Table S6.** Percentage increase (95% CI) of each adiposopathic marker for overweight (**BMI 25-29.9 kg/m^2^**) and obese (**BMI 30.0 kg/m^2^ or more)** vs not overweight **(BMI < 25.0 kg/m^2^)**, fitted separately for ages 43, 53 and 60-64 years.

|  | **Adipokines** | | **Inflammatory markers** | | **Endothelial markers** | | |
| --- | --- | --- | --- | --- | --- | --- | --- |
|  |  | |  | |  | | |
| **Overweight** | Adiponectin | Leptin | CRP | IL-6 | E-selectin | t-PA | vWF |
| **43 years** |  |  |  |  |  |  |  |
| Not overweight | - | - | **-** | - | - | - | - |
| Overweight | -15.5 (-22.3, -8.7) | 45.1 (37.7, 52.5) | 28.9 (19.9, 37.9) | 20.0 (12.8, 27.1) | 6.7 (2.0, 11.4) | 13.6 (7.3, 19.9) | 0.3 (-5.4, 6.0) |
| Obese | -34.7 (-45.5, -23.9) | 98.8 (87.1, 110.5) | 51.6 (37.3, 66.0) | 42.0 (30.7, 53.3) | 18.1 (10.7, 25.5) | 17.3 (7.3, 27.3) | 15.2 (6.2, 24.2) |
| **53 years** |  |  |  |  |  |  |  |
| Not overweight | - | - | - | - | - | - | - |
| Overweight | -25.4 (-32.3, -18.4) | 52.3 (45.0, 59.6) | 28.1 (18.8, 37.3) | 21.4 (14.1, 28.8) | 5.9 (1.0, 10.8) | 19.9 (13.4, 26.5) | -1.1 (-6.9, 4.8) |
| Obese | -43.0 (-51.5, -34.6) | 106.8 (98.0, 115.6) | 49.0 (37.8, 60.2) | 41.5 (32.6, 50.4) | 17.0 (11.1, 22.9) | 29.2 (21.2, 37.1) | 3.8 (-3.2, 10.9) |
| **60-64 years** |  |  |  |  |  |  |  |
| Not overweight | - | - | - | - | - | - | - |
| Overweight | -26.1 (-33.2, -19.0) | 64.6 (57.9, 71.2) | 24.4 (15.0, 37.8) | 15.4 (7.9, 22.9) | 8.2 (3.3, 13.1) | 23.0 (16.4, 29.6) | 1.6 (-4.5, 7.6) |
| Obese | -41.5 (-49.3, -33.7) | 128.9 (121.6, 136.3) | 57.1 (46.8, 67.5) | 42.2 (33.9, 50.5) | 24.4 (18.9, 29.8) | 38.2 (31.0, 45.4) | 10.0 (3.4, 16.6) |

**Table S7.** P-values for gender interactions with overweight status, fitted separately for each adiposopathic marker and age.

|  | **Adipokines** | | **Inflammatory markers** | | **Endothelial markers** | | |
| --- | --- | --- | --- | --- | --- | --- | --- |
|  |  | |  | |  | | |
| **Overweight** | Adiponectin | Leptin | CRP | IL-6 | E-selectin | t-PA | vWF |
| 2 years | 0.63 | 0.57 | 0.47 | 0.16 | **0.01** | 0.54 | 0.15 |
| 4 years | 0.20 | 0.90 | 0.11 | 0.80 | 0.74 | 0.43 | 0.51 |
| 6 years | 0.29 | 0.11 | 0.29 | 0.37 | 0.36 | 0.26 | 0.60 |
| 7 years | 0.16 | 0.10 | 0.87 | 0.50 | 0.95 | 0.30 | 0.86 |
| 11 years | 0.61 | 0.23 | 0.93 | 0.41 | 0.79 | 0.73 | 0.18 |
| 15 years | 0.61 | 0.73 | 0.09 | **0.03** | 0.19 | 0.58 | 0.37 |
| 20 years | 0.69 | 0.79 | 0.39 | **0.01** | 0.23 | 0.13 | 0.35 |
| 26 years | 0.90 | 0.91 | **0.01** | 0.07 | 0.20 | 0.80 | 0.10 |
| 36 years | 0.10 | 0.09 | **0.01** | **0.02** | 0.74 | 0.75 | 0.66 |
| 43 years | 0.75 | 0.51 | 0.36 | 0.07 | 0.82 | 0.95 | 0.36 |
| 53 years | 0.97 | 0.47 | 0.67 | 0.07 | 0.39 | 0.58 | 0.42 |
| 63 years | 0.58 | **<0.01** | **0.02** | **0.01** | 0.72 | 0.90 | 0.95 |

**Table S8.** Percentage increase (95% CI) of each inflammatory marker for overweight (BMI>=25 kg/m^2^) vs not overweight (BMI<25), fitted separately for each age and sex (n=1784).

|  | **Age- and sex-adjusted** | | | | **+ Adjustment for overweight at 60-64 years** | | | |
| --- | --- | --- | --- | --- | --- | --- | --- | --- |
|  | **C-reactive protein** | | **Interleukin-6** | | **C-reactive protein** | | **Interleukin-6** | |
|  |  | |  | |  |  |  |  |
| **Overweight** | Females | Males | Females | Males | Females | Males | Females | Males |
| 2 years | 8.1 (-5.4, 23.6) | **17.0 (2.1, 34.1)** | 8.1 (-5.4, 23.6) | **17.0 (2.1, 34.1)** | 8.5 (-4.7, 23.4) | **16.7 (1.9, 33.7)** | -0.4 (-10.5, 10.9) | **11.5 (0.2, 24.0)** |
| 4 years | -10.2 (-22.9, 4.7) | 7.2 (-8.1, 25.0) | -10.2 (-22.9, 4.7) | 7.2 (-8.1, 25.0) | -11.5 (-23.8, 2.8) | 7.4 (-7.8, 25.1) | -1.1 (-12.6, 11.8) | 2.6 (-9.0, 15.8) |
| 6 years | -9.7 (-25.5, 9.6) | 6.1 (-14.6, 31.9) | -9.7 (-25.5, 9.6) | 6.1 (-14.6, 31.9) | -13.1 (-28.0, 4.8) | 3.6 (-16.5, 28.6) | -5.3 (-18.9, 10.6) | 7.4 (-9.5, 27.5) |
| 7 years | -0.8 (-19.4, 22.2) | -4.1 (-27.0, 26.1) | -0.8 (-19.4, 22.2) | -4.1 (-27.0, 26.1) | -3.8 (-21.4, 17.8) | -9.2 (-30.9, 19.2) | -14.6 (-27.6, 0.8) | -6.2 (-24.4, 16.4) |
| 11 years | 8.9 (-11.8, 34.6) | 7.5 (-16.9, 39.1) | 8.9 (-11.8, 34.6) | 7.5 (-16.9, 39.1) | -1.0 (-19.7, 22.0) | 2.7 (-20.5, 32.8) | 1.7 (-14.1, 20.4) | 20.4 (-1.4, 47.0) |
| 15 years | **43.3 (16.1, 76.8)** | 6.5 (-18.0, 38.3) | **43.3 (16.1, 76.8)** | 6.5 (-18.0, 38.3) | **29.3 (4.9, 59.4)** | 0.7 (-22.3, 30.5) | **34.1 (13.5, 58.5)** | 5.5 (-14.2, 29.7) |
| 20 years | **36.7 (11.9, 67.1)** | **21.9 (0.6, 47.7)** | **36.7 (11.9, 67.1)** | **21.9 (0.6, 47.7)** | 19.4 (-2.2, 45.6) | 14.9 (-5.3, 39.5) | **26.6 (7.7, 48.9)** | -0.3 (-14.4, 16.2) |
| 26 years | **51.9 (27.6, 80.7)** | 14.2 (-1.5, 32.3) | **51.9 (27.6, 80.7)** | 14.2 (-1.5, 32.3) | **31.9 (10.6, 57.2)** | 6.8 (-8.2, 24.3) | **20.7 (4.5, 39.6)** | 10.3 (-1.8, 23.9) |
| 36 years | **51.3 (31.6, 74.0)** | **18.9 (5.0, 34.5)** | **51.3 (31.6, 74.0)** | **18.9 (5.0, 34.5)** | **32.5 (14.7, 53.2)** | 10.8 (-3.1, 26.6) | **20.8 (7.4, 35.8)** | 8.7 (-2.1, 20.7) |
| 43 years | **46.8 (30.6, 65.0)** | **34.9 (19.7, 52.0)** | **46.8 (30.6, 65.0)** | **34.9 (19.7, 52.0)** | **23.9 (9.1, 40.7)** | **28.6 (12.0, 47.5)** | **22.0 (10.1, 35.3)** | **19.3 (7.1, 32.8)** |
| 53 years | **43.8 (28.3, 61.2)** | **39.1 (22.2, 58.4)** | **43.8 (28.3, 61.2)** | **39.1 (22.2, 58.4)** | **15.2 (0.0, 32.8)** | **34.4 (13.3, 59.3)** | **21.4 (8.2, 36.1)** | **21.5 (6.1, 39.1)** |
| 63 years | **59.4 (42.3, 78.6)** | **29.0 (13.0, 47.3)** | **59.4 (42.3, 78.6)** | **29.0 (13.0, 47.3)** | **-** | **-** | **-** | **-** |

**Table S9.** Percentage increase (95% CI) of each adiposopathic marker for overweight (BMI>=25 kg/m^2^) vs not overweight (BMI<25), fitted separately for each age (n=1784, using multiple imputation).

|  | **Adipokines** | | **Inflammatory markers** | | **Endothelial markers** | | |
| --- | --- | --- | --- | --- | --- | --- | --- |
|  |  | |  | |  | | |
| **Overweight** | Adiponectin | Leptin | CRP | IL-6 | E-selectin | t-PA | vWF |
| 2 years | 6.0 (-2.1, 12.9) | -6.8 (-42.2, 1.4) | **11.8 (2.0, 22.5)** | 4.7 (-2.7, 12.6) | 1.8 (-2.9, 6.7) | 2.9 (-3.6, 9.9) | 2.4 (-3.6, 8.9) |
| 4 years | -0.4 (-8.1, 8.0) | -6.3 (-15.0, 3.3) | -1.0 (-10.9, 10.0) | 1.5 (-6.7, 10.5) | -0.4 (-5.9, 5.4) | 0.1 (-7.0, 7.8) | -4.2 (-10.4, 2.4) |
| 6 years | -1.8 (-12.2, 9.7) | -3.3 (-14.8, 9.8) | -5.0 (-17.5, 9.4) | 1.7 (-9.1, 13.8) | -3.0 (-9.9, 4.4) | -2.7 (-12.0, 7.5) | **10.0 (0.7, 20.1)** |
| 7 years | 2.6 (-9.3, 16.0) | 4.4 (-9.7, 20.7) | -1.4 (-16.0, 15.7) | -5.7 (-17.3, 7.5) | 1.0 (-7.4, 10.2) | -2.6 (-13.2, 9.3) | -2.6 (-12.1, 7.9) |
| 11 years | **-13.6 (-23.3, -23.0)** | **46.6 (27.6, 68.5)** | 6.9 (-8.8, 25.2) | **13.8 (0.3, 29.2)** | 3.6 (-4.8, 12.6) | 10.2 (-1.2, 22.9) | 1.4 (-7.9, 11.7) |
| 15 years | -8.0 (-18.5, 3.8) | **45.3 (26.9, 66.3)** | **21.7 (4.4, 41.8)** | **20.9 (7.1, 36.6)** | 6.5 (-15.9, 15.4) | **12.5 (1.1, 25.1)** | 1.2 (-8.0, 11.3) |
| 20 years | **-10.3 (-18.9, -0.7)** | **43.6 (27.9, 61.2)** | **23.6 (8.2, 41.2)** | **17.5 (5.8, 30.5)** | **7.9 (0.4, 15.9)** | 6.2 (-3.1, 16.5) | 15.9 (-6.6, 10.3) |
| 26 years | **-10.9 (-18.0, -3.1)** | **67.0 (51.5, 84.1)** | **27.3 (14.0, 42.2)** | **20.9 (10.7, 32.1)** | **5.9 (0.0, 12.1)** | **8.5 (0.4, 17.3)** | 6.9 (-0.2, 14.5) |
| 36 years | **-16.4 (-22.0, -10.5)** | **71.0 (58.3, 84.6)** | **30.8 (19.4, 43.3)** | **21.7 (13.1, 30.8)** | **10.1 (4.4, 14.8)** | **16.1 (9.0, 23.7)** | 4.1 (-1.6, 10.2) |
| 43 years | **-18.4 (-23.4, -13.2)** | **77.9 (66.0, 90.6)** | **40.0 (29.0, 52.0)** | **28.4 (20.2, 37.2)** | **10.7 (6.0, 15.6)** | **17.0 (10.4, 23.9)** | 4.0 (-1.2, 9.6) |
| 53 years | **-26.5 (-31.0, -21.6)** | **102.6 (89.2, 117.1)** | **41.1 (29.6, 53.7)** | **30.9 (22.2, 40.2)** | **10.5 (5.6, 15.6)** | **26.3 (19.1, 34.0)** | 1.3 (-4.0, 6.9) |
| 63 years | **-27.7 (-32.2, -22.8)** | **146.3 (130.5, 163.1)** | **45.0 (33.0, 58.1)** | **29.2 (20.5, 38.5)** | **16.1 (10.9, 21.4)** | **33.6 (25.8, 41.8)** | 5.0 (-0.6, 10.9) |
